
JOURNAL INFORMATION
==============================
NLM Title Abbreviation: City Soc (Wash)
Journal ID: City Soc (Wash)
NLM Journal ID: 101561710
Journal ID: CISO

City & Society (Washington, D.C.)

ISSN: 0893-0465
EISSN: 1548-744X

ARTICLE INFORMATION
==============================
PMCID: PMC7361612
PMID: 32836784
DOI: 10.1111/ciso.12310
Article ID: CISO12310
Article version: 1
Subjects: Original Article, COVID‐19 City Dispatches

A Divided City in a Time of Pandemic: Dispatches from Doha

Chandra Uday 1
Promodh Irene 1
1 Georgetown University in Qatar
Electronic publication date: 2020 Jun 22
Print publication date: 2020 Aug
Volume: 32
Issue: 2
Electronic Location ID: 10.1111/ciso.12310
Copyright: © 2020 The Authors. City & Society published by Wiley Periodicals LLC on behalf of American Anthropological Association.
License: This is an open access article under the terms of the http://creativecommons.org/licenses/by/4.0/ License, which permits use, distribution and reproduction in any medium, provided the original work is properly cited.
License URL: https://creativecommons.org/licenses/by/4.0/

Figure count: 0
Table count: 0
Page count: 16

source-schema-version-number: 2.0
cover-date: August 2020
details-of-publishers-convertor: Converter:WILEY_ML3GV2_TO_JATSPMC version:6.1.7 mode:remove_FC converted:13.07.2022

==============================

CITY & SOCIETY

*City & Society*

### A Divided City in a Time of Pandemic: Dispatches from Doha

*Uday Chandra and Irene Promodh, Georgetown University in Qatar*

Doha, once a sleepy Indian Ocean port and now a symbol of Qatar's rise as a petro-state, is a city divided between its wealthy citizens and resident aliens and the migrant workers that serve them. The city's spatial divisions are rooted in its transoceanic history as much as its makeover as an urban experiment in authoritarian modernism (Hashim, Irazábal, and Byrum 2010). As Qatar's monarchical regime liberalized its economy with an eye to the post-hydrocarbon future (Nonneman 2006), Doha came to be defined by imposing edifices towering over awestruck subjects and a lack of public spaces and street corners in which different strata of society can mingle or gather freely. With the arrival of COVID-19, the well-planned residential neighborhoods of wealthy citizens and expatriates seem starkly separated from those of the working poor even as their dependence on each other is arguably greater than ever before. This deeply divided city, wrestling with the politics of contagion, shows how logics of exclusion and interdependence are, paradoxically, intertwined inextricably.

© 2020 The Authors. City & Society published by Wiley Periodicals LLC on behalf of American Anthropological Association. This is an open access article under the terms of the Creative Commons Attribution License, which permits use, distribution and reproduction in any medium, provided the original work is properly cited. DOI:10.1111/ciso.12310

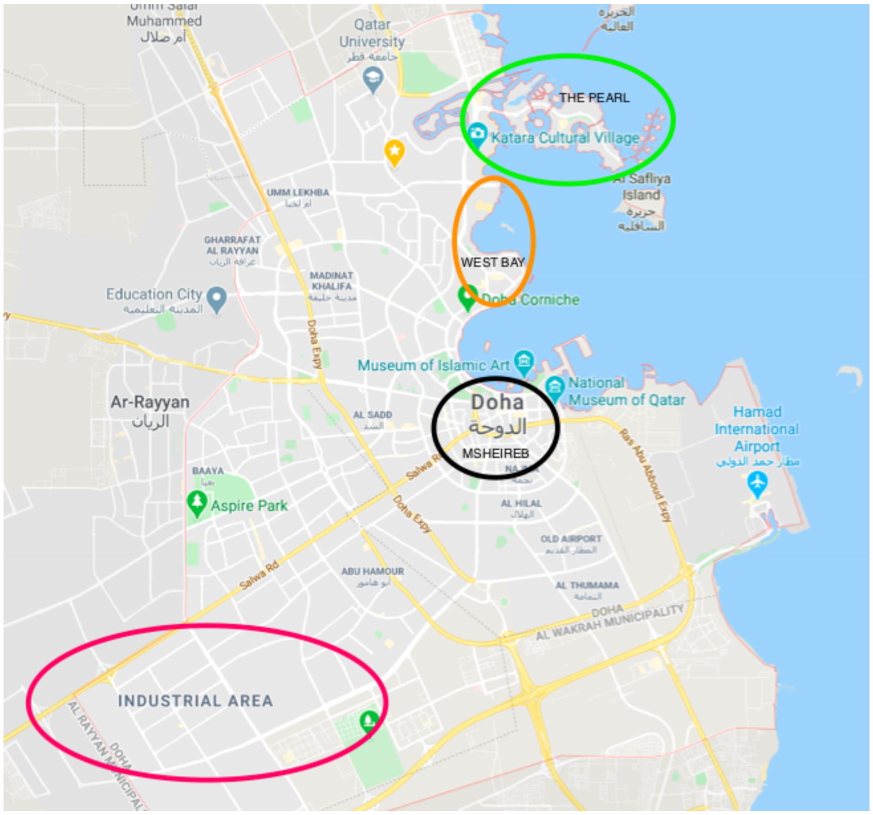

Fig. 1: Map of Doha; encircled districts: the Industrial Area, Msheireb, West Bay, and the Pearl (Date accessed: 5 June 2020)

In Doha, as in other Persian Gulf capitals, the construction of skyscrapers and sustainable living spaces for the well-to-do and the city's new working-class neighborhoods (*fireej* in the local Khaleeji dialect) occurred simultaneously. Old fishing villages and the colonial-era *medina* (Azzali 2019) gave way to projects of urban renewal such as the Msheireb Downtown Doha project, the waterfront business district in West Bay, and the Pearl-Qatar island. These innovative experiments in urban living, showcasing buildings designed by I.M. Pei, Jean Nouvel, Zaha Hadid and others, blend a homegrown neo-Bedouin aesthetic with the latest architectural trends (Furlan 2016; Furlan et al 2019). The functional aesthetics of Doha's working-class

© 2020 The Authors. City & Society published by Wiley Periodicals LLC on behalf of American Anthropological Association. This is an open access article under the terms of the Creative Commons Attribution License, which permits use, distribution and reproduction in any medium, provided the original work is properly cited. DOI:10.1111/ciso.12310

neighborhoods, especially the Industrial Area that houses the majority of the city's two million blue-collar workers, contrast sharply with the glamorous new residential districts for well-heeled expats and citizens (Ibrahim et al. 2019). Working-class neighborhoods are almost exclusively male domains, men comprising roughly three-fourths of the city's population of 640 million, mostly poor Asian and African migrants from across the Indian Ocean world. The Industrial Area as a separate new residential district of Doha, complete with its own mall, movie theater, and cricket stadium, is a world away from the dazzling lights of West Bay.

The arrival of COVID-19 in Doha at the end of February was met with a sense of nonchalance that soon gave way to fear and confusion. Few appeared perturbed when a handful of Qatari citizens were evacuated from Iran and quarantined in a plush hotel in West Bay. By mid-March, however, malls, restaurants, banks, and public transport shut down as did flights into Doha. Social gatherings were prohibited as mosques, public parks, and beaches closed. A part of the Industrial Area was completely closed off. Doctors and employers were required by law to report positive tests or face fines of up to 200,000 riyals (USD 55,000) or imprisonment. Fourteen citizens who had contracted the virus during their stay in Iran had signed pledges to self-quarantine at home, but when they failed to do so, state television named and shamed them before arresting nine of them. While white-collar "expats" began referring to Doha as "home" as they chose to stay behind in lavish employer-provided apartments, blue-collar "migrants" faced a new dilemma: to go to work and potentially ruin one's physical health and long-term economic prospects or not go to work and deplete any savings to remit home and face deprivation and hunger. In late April, the government introduced a new app named Ehtaraz that used QR color

© 2020 The Authors. City & Society published by Wiley Periodicals LLC on behalf of American Anthropological Association. This is an open access article under the terms of the Creative Commons Attribution License, which permits use, distribution and reproduction in any medium, provided the original work is properly cited. DOI:10.1111/ciso.12310

codes to distinguish between virus-free individuals ("green") and those with suspected symptoms or in contact with positive cases ("grey") from those who tested positive for COVID-19 ("red") and those in quarantine ("yellow"). Qatar never declared a formal lockdown and the death toll in Qatar is among the lowest in the world, but masks and the Ehtaraz app are mandatory with steep fines of up to 200,000 riyals (USD 55,000) or imprisonment as penalties for violations.

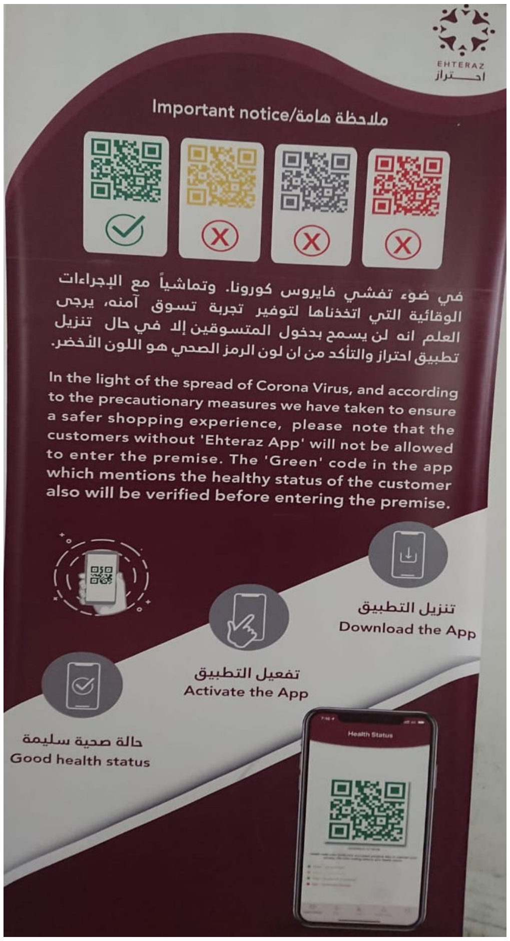

Fig. 2: Guidelines on the use of the mandatory Ehtaraz app at a supermarket entrance (Photo credit: Uday Chandra, 6 June 2020)

© 2020 The Authors. City & Society published by Wiley Periodicals LLC on behalf of American Anthropological Association. This is an open access article under the terms of the Creative Commons Attribution License, which permits use, distribution and reproduction in any medium, provided the original work is properly cited. DOI:10.1111/ciso.12310

### The Spatial Politics of Contagion

With the onset of COVID-19, access to masks, gloves, and sanitizers as well as the ability to distance oneself from others took on an uneven distribution across Doha's spatial divides. In the Industrial Area, migrant workers expressed concerns over poor standards of hygiene and sanitation in overcrowded living quarters and communal dining spaces where social-distancing and mask-wearing norms were flouted routinely on the grounds of being impractical. Although supermarkets were stocked with masks and hand sanitizers, most migrant workers, temporarily unemployed and confined to their residential compounds, found it difficult to spare 40 riyals for 20 masks on salaries of 800 riyals/month (QQ1, interview, 7 May, 2020). Given the impossibility of social-distancing in their dormitories, purchasing and wearing masks also seemed redundant to many (QQ2, interview, 7 May, 2020). In addition, many were not allowed by their employers to leave their living quarters. As such, even for those who *could* afford masks, their confinement to their Industrial Area compounds prevented them from going out to buy them (QQ2, interview, 7 May, 2020). This was effectively a part of the city that has been marked "red" as either infected or susceptible to infection, though no such official zoning norms exist.

© 2020 The Authors. City & Society published by Wiley Periodicals LLC on behalf of American Anthropological Association. This is an open access article under the terms of the Creative Commons Attribution License, which permits use, distribution and reproduction in any medium, provided the original work is properly cited. DOI:10.1111/ciso.12310

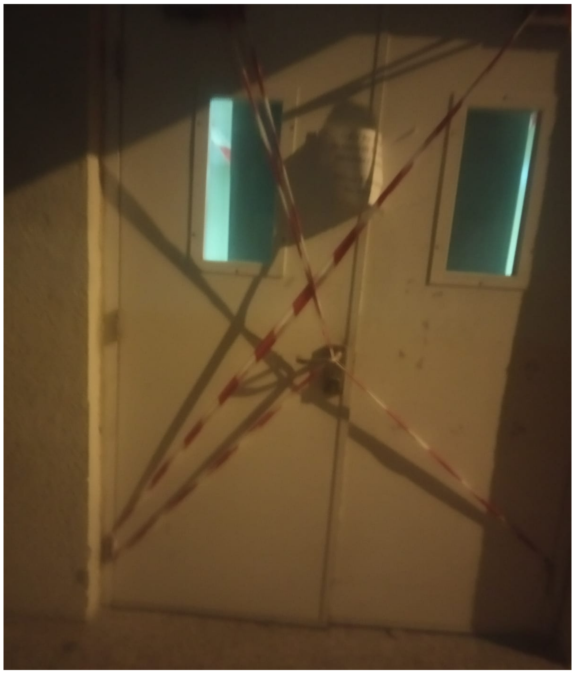

Fig. 3: Doors sealed in an Asian City building in the Industrial Area to restrict the movements of residents (Photo credit: QQ3, 3 June 2020)

By contrast, the West Bay waterfront district and the Pearl Doha, inhabited mostly by white-collar expats and citizens, operated as a de facto green zone. Mask-wearing residents in their branded sports gear went about their daily exercise regime, whether cycling, running, or walking. Set apart from the rest of the city as "safe" or infection-free spaces, these residential districts served as ideal spaces for families and friends to gather during the pandemic even when they violated official regulations in place. It was not uncommon to hear complaints about an influx of "outsiders" into these "safe" spaces in the evenings. The occasional police patrol served as a reminder of the state's presence, but as long as someone wore masks and, later, showed their "green" status on the Ehtaraz app, they could move freely throughout these spaces. Residents in these upscale residential neighbourhoods were more concerned with their privacy than by the

© 2020 The Authors. City & Society published by Wiley Periodicals LLC on behalf of American Anthropological Association. This is an open access article under the terms of the Creative Commons Attribution License, which permits use, distribution and reproduction in any medium, provided the original work is properly cited. DOI:10.1111/ciso.12310

risk of infection. It was hard to tell that this was a country with the highest per capita number of confirmed cases in the world. No city had a higher concentration of COVID-19 anywhere.

Much like residential neighborhoods, grocery stores and supermarkets became microcosms that reflected the spatial politics of the city. Whereas wealthy expats and citizens were highly mobile and capable of visiting any supermarket, this was not necessarily the case for the majority of the city's residents, especially the two million or so blue-collar migrant workers. Grocery stores and supermarkets operated as exclusive spaces for the former even as a small selection of the latter group were employed to keep these establishments open. Measures to ensure hygiene and safe shopping changed over time from the mandatory use of hand sanitizers in March to the compulsory use of masks, gloves, and, eventually, the Ehtaraz app. However, certain regulations have been in place throughout. For instance, some categories of persons, most notably children under the age of 12, pregnant women, adults aged over 55, and individuals with chronic illnesses, were advised against visiting grocery stores. Likewise, security guards, migrants from a range of Asian and African countries along the Indian Ocean rim, were entrusted with conducting temperature checks, and subsequently, QR code checks. The entrances to supermarkets, meeting places for people across the city's social divides, became highly contentious spots in which both the enforcement and evasion of public regulations were highly visible. At the same time, public health notices about COVID-19 and the latest regulations concerning it were displayed prominently at the entrances and key areas of the supermarkets, but in the aisles and checkout lines, shoppers themselves did not always adhere to social-distancing norms. The most exclusive grocery stores, nonetheless, sought to present themselves as the safest places to shop in the city.

© 2020 The Authors. City & Society published by Wiley Periodicals LLC on behalf of American Anthropological Association. This is an open access article under the terms of the Creative Commons Attribution License, which permits use, distribution and reproduction in any medium, provided the original work is properly cited. DOI:10.1111/ciso.12310

For instance, Monoprix, touted as the most sanitized and hygienic place to shop in Qatar, started sanitizing shopping carts individually via special docks. The aesthetics of plenitude suffused these stores. Contrasting visibly with social media images of empty grocery store shelves and stockpiling from Europe and the United States, visuals of a wide and plentiful array of grocery items, particularly food supplies, were most striking in even the humblest grocery store. The politics of food security at work here, as economists have explained, goes back to Qatar's response to the ongoing tensions with neighboring Saudi and Emirati states since June 2017. The supermarket became, in sum, a metonym of the thriving nation and its capital city, albeit in ways that reflected its spatial politics.

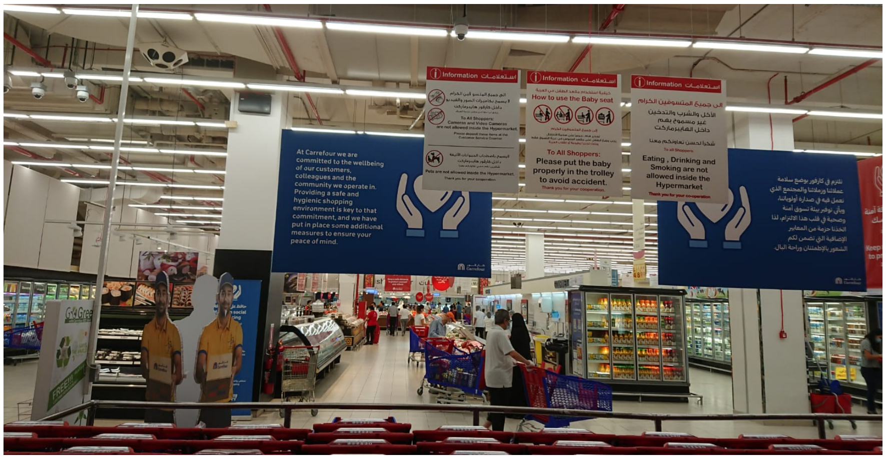

Fig. 4: Inside a West Bay branch of Carrefour supermarket (Photo credit: Uday Chandra, 6 June 2020)

© 2020 The Authors. City & Society published by Wiley Periodicals LLC on behalf of American Anthropological Association. This is an open access article under the terms of the Creative Commons Attribution License, which permits use, distribution and reproduction in any medium, provided the original work is properly cited. DOI:10.1111/ciso.12310

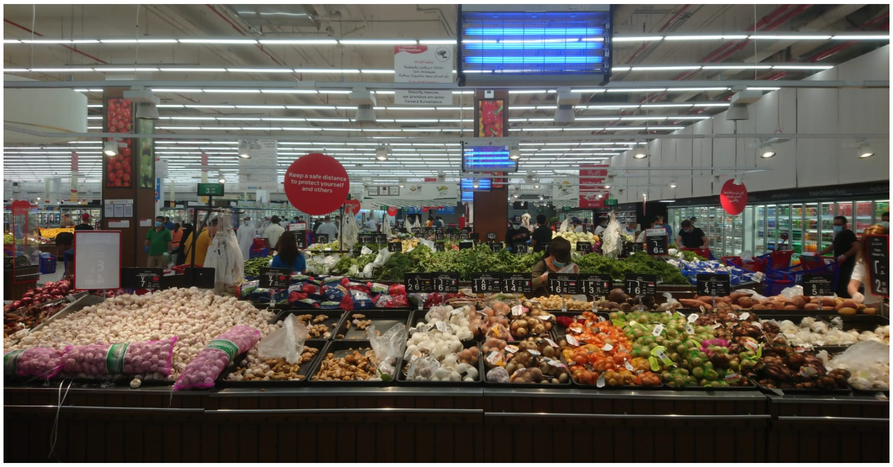

Fig. 5: Well-stocked supplies of fresh produce in a West Bay supermarket (Photo credit: Uday Chandra, 6 June 2020)

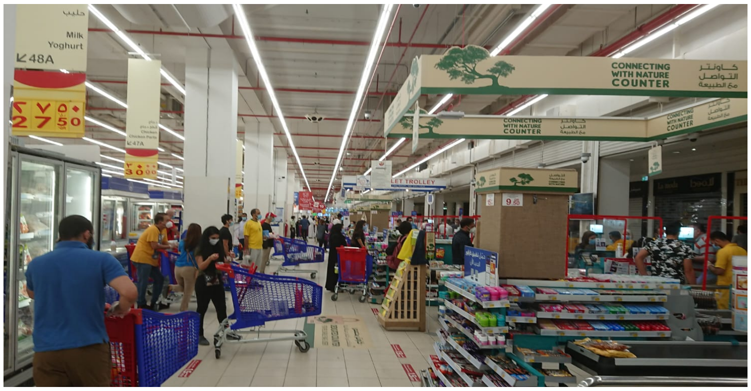

Fig. 6: Grocery checkout lines that ignore social-distancing guidelines (Photo credit: Uday Chandra, 6 June 2020)

On the outskirts of Doha, the underbelly of the thriving city was revealed. Blue-collar migrants housed in Industrial Area's Asian City seemingly operated according to a different set of norms from the city's wealthier denizens. Social-distancing was more or less impossible in workers' accommodations that housed typically four to five, sometimes even eight to ten males, in a single dormitory. Shared bathrooms, densely-crowded dining spaces, and the constant inflow and outflow of residents working at construction sites greatly exacerbated the risk of contagion. From

© 2020 The Authors. City & Society published by Wiley Periodicals LLC on behalf of American Anthropological Association. This is an open access article under the terms of the Creative Commons Attribution License, which permits use, distribution and reproduction in any medium, provided the original work is properly cited. DOI:10.1111/ciso.12310

first-hand accounts of migrants in Asian City, we learned that, contrary to government statements, no-cost gloves, masks, and sanitizers were largely unavailable for their use. Some construction workers wore masks and gloves they had received at their worksites in their living quarters and dining spaces (QQ1, interview, 7 May, 2020). For those without jobs, however, the lack of masks, gloves, sanitizers, *and* social-distancing in overcrowded dormitories translated into poor sanitary standards. Not only did this state of affairs result in a rapid spread of infection in the neighborhood, but it greatly increased anxieties over lives and livelihoods. These anxieties were heightened further with the quarantining of a part of the Industrial Area, an area that was already out of bounds for the rest of Doha's denizens. The spatial politics of the city thus began to reveal itself in the city's most densely populated and affected neighborhood.

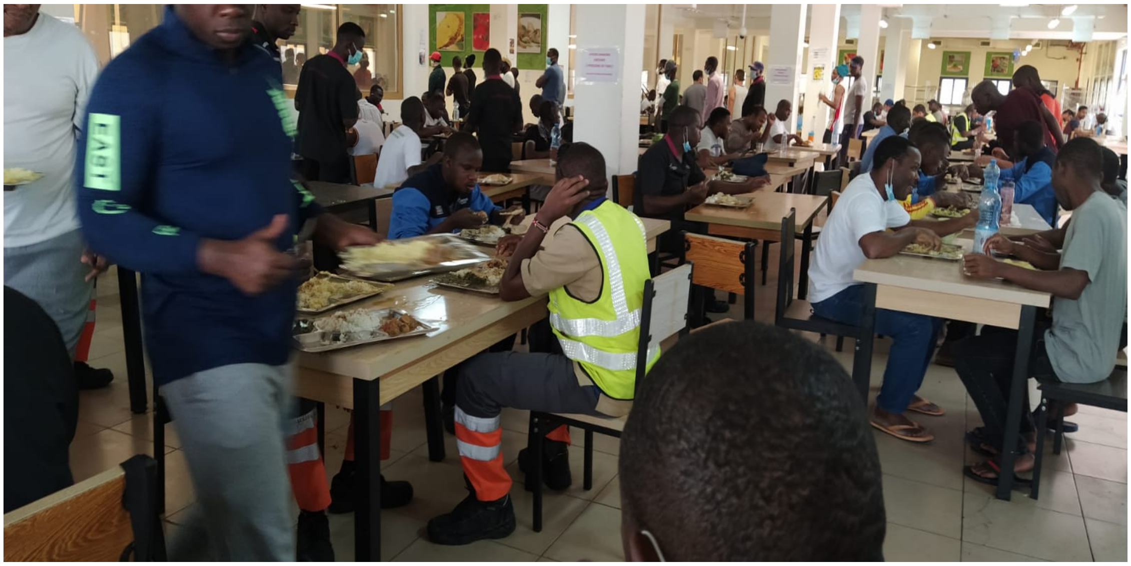

Fig. 7: No masks or social distancing at a communal dining space in Asian City, Industrial Area (Photo credit: QQ4, 2 JUNE 2020)

© 2020 The Authors. City & Society published by Wiley Periodicals LLC on behalf of American Anthropological Association. This is an open access article under the terms of the Creative Commons Attribution License, which permits use, distribution and reproduction in any medium, provided the original work is properly cited. DOI:10.1111/ciso.12310

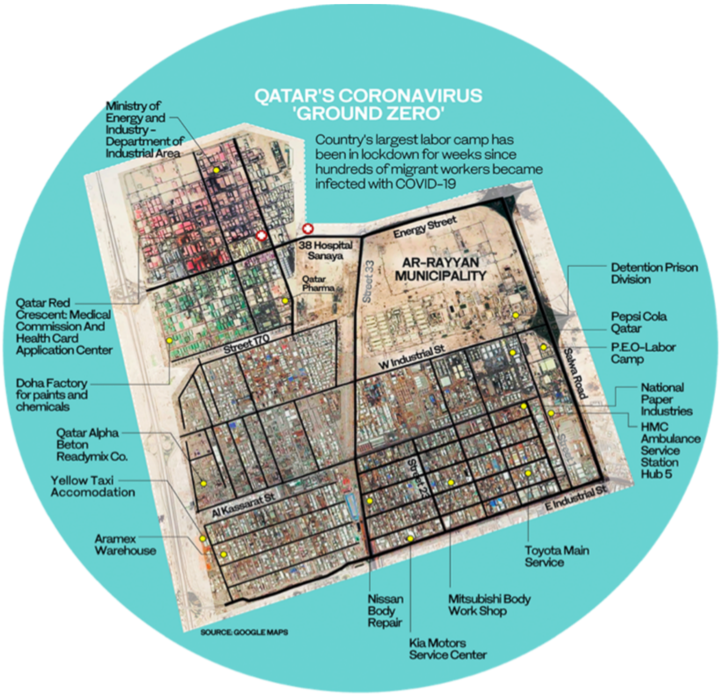

Fig. 8: A satellite image of Qatar's Industrial Area, previously under lockdown (ArabNews, date accessed: 5 June 2020)

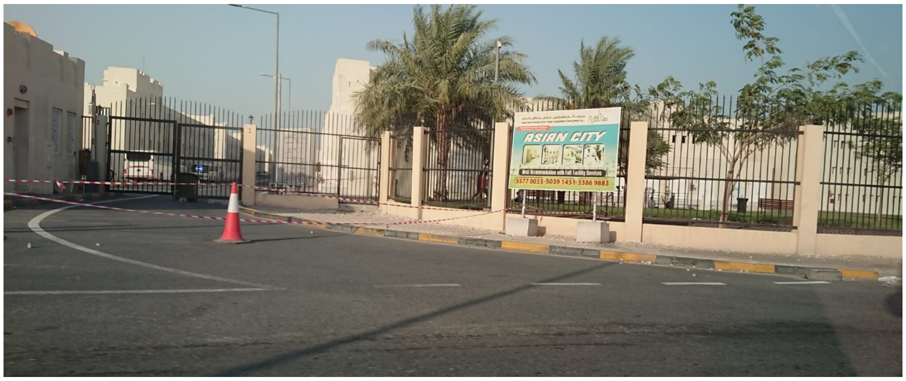

Fig. 9: An entrance to Asian City in the Industrial Area (Photo credit: Uday Chandra, 5 June 2020)

© 2020 The Authors. City & Society published by Wiley Periodicals LLC on behalf of American Anthropological Association. This is an open access article under the terms of the Creative Commons Attribution License, which permits use, distribution and reproduction in any medium, provided the original work is properly cited. DOI:10.1111/ciso.12310

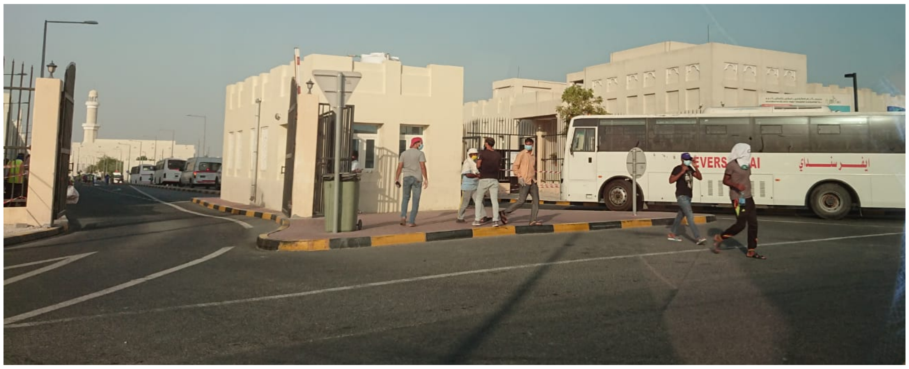

Fig. 10: Another entrance to Asian City in the Industrial Area (Photo credit: Uday Chandra, 5 June 2020)

Construction sites across the city were also states of exception within Doha's quarantine regime. Each day throughout the pandemic, hundreds of construction workers were transported in overcrowded buses from the Industrial Area to construction sites across the city. In these buses, neither masks nor social-distancing norms were visible, much to the dismay and chagrin of the workers. On their work sites, however, construction workers were, as required by the government and construction firms, provided face masks and issued social-distancing guidelines. The responsibility for following these guidelines, of course, rested wholly on the workers themselves. In hot weather, it was not unusual to spot workers with masks lowered or without masks altogether along highways or outside construction sites. Construction sites themselves, uninhabited and still in scaffolds, were relatively isolated work spaces from their urban surroundings. In pursuit of jobs and a steady income amidst mass layoffs across the city, construction workers effectively found themselves placed *outside* Doha's quarantine regime with its performative politics of visible mask-wearing and social-distancing. At the same time, their state of exception ensured that Qatar's high-modernist architectural projects went ahead as planned, particularly in preparation for the 2022 FIFA World Cup.

© 2020 The Authors. City & Society published by Wiley Periodicals LLC on behalf of American Anthropological Association. This is an open access article under the terms of the Creative Commons Attribution License, which permits use, distribution and reproduction in any medium, provided the original work is properly cited. DOI:10.1111/ciso.12310

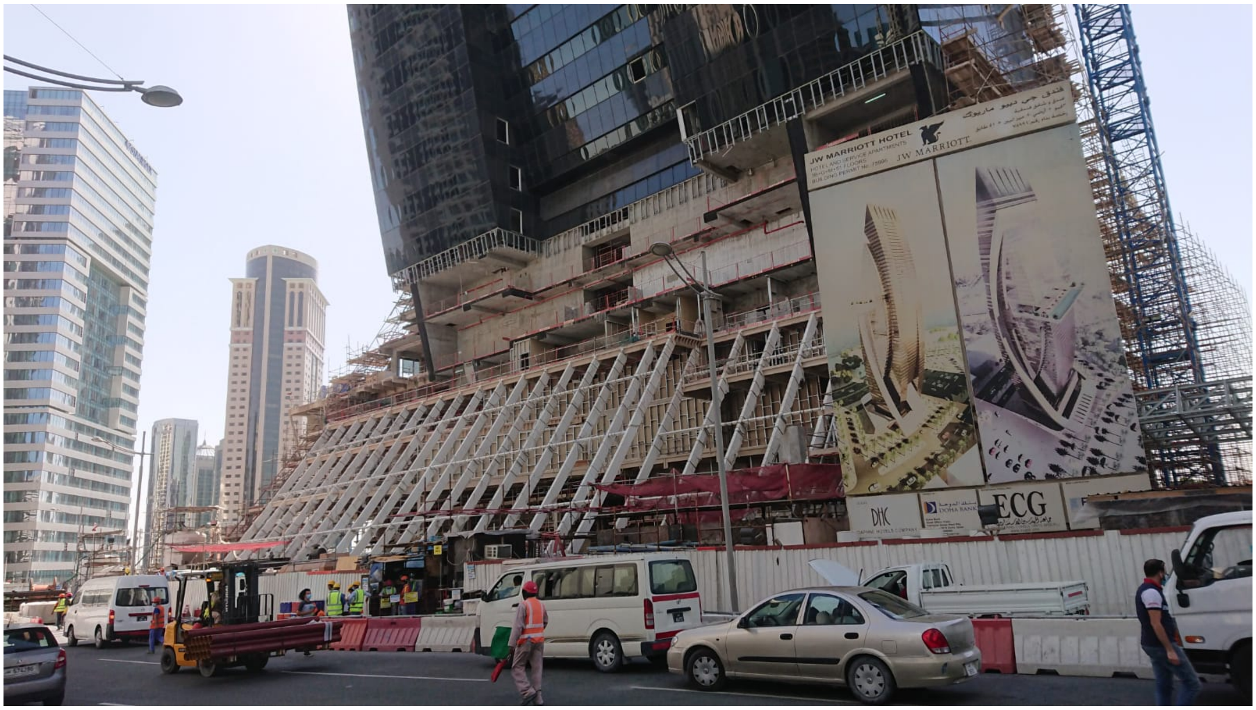

Fig. 11: Migrant workers continue to work at a West Bay construction site during the pandemic (Photo credit: Uday Chandra, 5 June 2020)

Much like construction workers, “gig workers” continued to leave their homes for work each day, but their work required them to traverse Doha’s spatial divides during the pandemic. The most prominent gig workers in the city were those hired for on-demand food deliveries as well as Uber drivers. As the city’s wealthy denizens, working from home or not at all, grew increasingly wary of in-person shopping and yearned to go back to their dining-out routines, grocery and food delivery apps soared in popularity. Itinerant migrant workers, usually from East African and South Asian countries, needed jobs in a time of financial precarity, and turned in large numbers to delivering groceries and cooked food. At the same time, Talabat, a regional delivery app

© 2020 The Authors. City & Society published by Wiley Periodicals LLC on behalf of American Anthropological Association. This is an open access article under the terms of the Creative Commons Attribution License, which permits use, distribution and reproduction in any medium, provided the original work is properly cited. DOI:10.1111/ciso.12310

popular among Qatar's expats and citizens, adapted to the new circumstances by advertising their unique healthy and safety measures, including contactless, safe delivery across the city. Talabat also no longer featured primarily fast food and medium-range restaurants, but became a portal to the city's fine dining scene. In turn, the city's five-star hotels and independent high-end restaurants became increasingly dependent on the gig economy, most notably apps such as Talabat, to stay afloat financially. Migrant workers picking up and dropping off food and groceries gradually became the most visible presence on Doha's streets in March and April. By contrast, Uber drivers grew restive without a regular flow of passengers and their earnings plummeted with every passing week. Our ethnographic data suggest that Uber drivers' earnings dipped below 100 riyals (approx. USD 27) per day, somewhere between half to a third of their typical levels. Many began to work shifts of 14 to 16-hours after the arrival of COVID-19, though passengers grew increasingly fearful of the closed, contagion-prone spaces of Uber taxis. Some drivers reported that their earnings peaked between 2 and 4 AM daily when passengers, particularly air hostesses and other cabin crew residing in West Bay and the Pearl, travelled to or from the airport. Both food delivery workers and Uber drivers, who routinely breach the spatial divides of the city in the course of their work, now found themselves the only ones doing so in a time of pandemic. Although both were seen by the city's wealthier residents as sources of infection, the effects on these two sets of workers differed radically. While Uber drivers grew anxious as the virus threatened their uncertain livelihoods by inducing a dearth of passengers, food delivery workers, whose numbers grew during the pandemic, risked daily exposure to the virus during their itinerant work, which, in fact, further reinforced Doha's spatial divides.

© 2020 The Authors. City & Society published by Wiley Periodicals LLC on behalf of American Anthropological Association. This is an open access article under the terms of the Creative Commons Attribution License, which permits use, distribution and reproduction in any medium, provided the original work is properly cited. DOI:10.1111/ciso.12310

### Retrospect and Prospect

In *Discipline and Punish*, Michel Foucault (1995: 195-200) contrasted the pre-modern state logic of excluding lepers from medieval European cities to the modern state's "panopticism" with regard to plague victims within quarantined urban spaces. For Foucault, the birth of the modern state was tied inextricably to the rise of a new disciplinary mechanism by which power flowed through multiple capillaries that suffused all individuals as state subjects. Our dispatches from Doha during the COVID-19 pandemic urge us to think with Foucault and, at the same time, rethink the modalities of power at work in our times. On the one hand, as we see in Doha, statist logics of exclusion and *quadrillage* can happily coexist with each other in the time of a pandemic. On the other hand, in a digital age, modern state power in Qatar, as elsewhere, is much less omniscient and all-encompassing than what Foucault proposed.

Doha's spatial divides long predated the arrival of COVID-19. Indeed, these divides are hardwired into the city's makeover from a colonial port city into a twenty-first century metropolis. Under a regime of authoritarian high modernism, the separation of the residential neighbourhoods of the well-to-do from the working poor is as striking as their dependence on each other. In this social setting, the liminality generated by a virus threatened to efface the city's social divides as white-collar expats faced the prospect of ending up in the same quarantine facilities as blue-collar migrants. Over time, however, the virus seemingly adapted itself to the city and effectively reinforced its spatial divides as much as their mutual dependence. Social exclusion and disciplining thus came to coexist with each other in much the same way as the city's diverse residents. The hybridity of modern state power in Doha mirrors by the mélange of

© 2020 The Authors. City & Society published by Wiley Periodicals LLC on behalf of American Anthropological Association. This is an open access article under the terms of the Creative Commons Attribution License, which permits use, distribution and reproduction in any medium, provided the original work is properly cited. DOI:10.1111/ciso.12310

lifeworlds and livelihoods that defy any singular logic. All of Doha's denizens are plague victims today, but only some are treated akin to lepers in medieval Europe. This is admittedly an authoritarian regime, but a soft one unlike those in neighboring Saudi Arabia and the United Arab Emirates, whose urban lockdowns, neoliberal economic reforms, and new fiscal arrangements follow a more punitive and unitary logic. Nor is it the case that Qatar seeks to drastically reduce its dependence on migrants as Kuwait has announced in an election year. A hybrid soft authoritarianism may shore up existing social hierarchies, but may also be more humane than democracies and nondemocracies that wish to keep out migrants altogether.

In the Qatari state's pronouncements since the onset of the pandemic, it has sought to assure citizens and non-citizens alike that all is well. We learned that hospital beds, medical equipment, sanitary supplies, and foodstocks were more than adequate to tide us through this crisis. A mix of officially-generated numbers and Twitter posts conveyed to us that the state's pastoral and disciplinary functions were more than capable of tackling the virus. However, with one in forty infected in the city, the pandemic has brought a sense of pandemonium rather than creating pandemia or a "rally around the flag" effect. Official statistics and pronouncements issued via state media have heightened this sense of pandemonium. The panoply of regulations that have emerged in the past three months, implemented unevenly across the city, have hardly alleviated anxieties. Across the social divides of the city, these regulations are flouted far more often than the state would care to admit. The wealthy shun masks to exercise outdoors in their safe spaces. The poor shun masks because they cannot afford to buy them, they simply do not receive them at their accommodations, and/or it is too hot to wear while working outdoors in the summer

© 2020 The Authors. City & Society published by Wiley Periodicals LLC on behalf of American Anthropological Association. This is an open access article under the terms of the Creative Commons Attribution License, which permits use, distribution and reproduction in any medium, provided the original work is properly cited. DOI:10.1111/ciso.12310

months. Norms of social-distancing are, similarly, observed as often as they are breached across the city, whether in supermarkets or on the streets. Ultimately, the reopening of offices and malls is accompanied by a sense of helplessness on the part of the state, which now accepts that the spread of the virus is more or less inevitable and face masks, hand sanitizers, and contact tracing are our only means of defense against an invisible enemy. Far from the Foucauldian depictions of the early modern leviathan, we are saddled with an anxious state whose writ wears thin on its befuddled subjects.

### References

Azzali, Simona. 2019. "Spaces of Mega Sporting Events versus Public Spaces". *The Journal of Public Space* 4 (2): 57-80.

Furlan, Raffaello. 2016. "Modern and Vernacular Settlements in Doha: An Urban Planning Strategy to Pursue Modernity and Consolidate Cultural Identity." *Arts and Social Sciences Journal* 7 (2).

Furlan, Raffaello, et al. 2019. "The Urban Regeneration of West-Bay, Business District of Doha (State of Qatar): A Transit-Oriented Development Enhancing Livability." *Journal of Urban Management* 8 (1): 126–44.

Foucault, Michel. 1995. *Discipline and Punish: The Birth of the Prison*. New York: Vintage Books.

Ibrahim, Hatem, et al. 2019. "Investigating Housing Distribution for the Expatriate Population in Doha." *Urban, Planning and Transport Research* 7 (1): 34–52.

Nonneman, Gerd. 2008. "Political Reform in the Gulf Monarchies: From Liberalisation to Democratisation? A Comparative Perspective." In E. Anoushiravan and S. Wright, *Reform in the Middle East Oil Monarchies*. Berkshire, UK: Ithaca Press.

© 2020 The Authors. City & Society published by Wiley Periodicals LLC on behalf of American Anthropological Association. This is an open access article under the terms of the Creative Commons Attribution License, which permits use, distribution and reproduction in any medium, provided the original work is properly cited. DOI:10.1111/ciso.12310
